# Thymic Lymphoepithelial Carcinoma Associated with Epstein-Barr Virus: Experiences and Literature Review

**DOI:** 10.3390/cancers13194794

**Published:** 2021-09-24

**Authors:** Naoko Ose, Sachi Kawagishi, Soichiro Funaki, Takashi Kanou, Eriko Fukui, Kenji Kimura, Masato Minami, Yasushi Shintani

**Affiliations:** 1 Department of General Thoracic Surgery, Osaka University Graduate School of Medicine, Suita-shi 565-0871, Osaka, Japan; funaki@thoracic.med.osaka-u.ac.jp (S.F.); tkanou@thoracic.med.osaka-u.ac.jp (T.K.); fukui@thoracic.med.osaka-u.ac.jp (E.F.); kenkimura@thoracic.med.osaka-u.ac.jp (K.K.); maminami@hp-op.med.osaka-u.ac.jp (M.M.); yshintani@thoracic.med.osaka-u.ac.jp (Y.S.); 2 Department of General Thoracic Surgery, Osaka International Cancer Institute, Chuo-ku, Osaka-shi 541-8567, Osaka, Japan; sachi.kawagishi@oici.jp

**Keywords:** thymus, lymphoepithelial carcinoma, Epstein-Barr virus, thymic epithelial tumor, thymic cancer, review

## Abstract

**Simple Summary:**

Thymic lymphoepithelial carcinoma (TLEC) is a rare primary thymic carcinoma. EBV infection has been reported among some individuals with TLEC tumor cells. Instances of EBV infection in other types of thymic epithelial tumor have been reported at lower rates, which suggests that EBV infection may have an important influence on the carcinogenesis of TLEC, though the etiology is unknown. Though there have been reports of thymic carcinoma including TLEC, there are few reports on the analysis of TLEC alone, and only case-reports were reported. We conducted this review by accumulating 58 cases in 34 reports to date. TLEC is a highly malignant tumor with poor prognosis, as affected patients have a median survival time of 22 months, according to 58 cases from the literature, while 5-year survival rate is 34.4%. Presently, prognosis is not considered to be affected by the presence or absence of EBV positivity.

**Abstract:**

Thymic lymphoepithelial carcinoma (TLEC) is a primary thymic carcinoma that accounts for about 14% of all thymic epithelial tumors and is classified into 14 types. The histological morphology is similar to lymphoepithelioma, a type of undifferentiated nasopharyngeal carcinoma. It has been reported that squamous carcinoma accounts for approximately 80% of thymic carcinoma, followed by TLEC, which accounts for 6%. TLEC has been reported to be associated with Epstein-Barr virus (EBV), with EBV infection in TLEC tumor cells first noted by Lyvraz et al. in 1985. Tumors shown to be EBV-positive are classified as TLEC if lymphoplasmacytic infiltration is lacking. However, only about 50% of the cases are positive for EBV, which is lower compared to nasopharyngeal lymphoepithelioma. Instances of EBV infection in other types of thymic epithelial tumor have been reported at lower rates, which suggests that EBV infection may have an important influence on the carcinogenesis of TLEC, though the etiology is unknown. TLEC is a highly malignant tumor with poor prognosis, as affected patients have a median survival time of 22 months, according to 58 cases from the literature, while the 5-year survival rate is 34.4%. Presently, prognosis is not considered to be affected by the presence or absence of EBV positivity.

## 1. Introduction

Thymic lymphoepithelial carcinoma (TLEC) is defined as a primary thymic undifferentiated or poorly differentiated squamous cell carcinoma or undifferentiated carcinoma, with significant prominent reactive lymphoplasmacytic infiltration identical to undifferentiated nasopharyngeal carcinoma [1,2]. Cancers in organs other than a nasopharynx, which show histological similarities to that of lymphoepithelioma, are called lymphoepithelioma-like carcinomas (LELC) or lymphoepithelial carcinomas (LEC). In both lymphoepithelioma and LEC, the EBV genome is detected in tumor cells, and EBV is closely associated with their tumorigenesis [3,4]. The EBV genome has been detected in 90% of nasopharyngeal lymphoepitheliomas and 10% of gastric cancers [3,4]. Thymic LEC has the capacity to metastasize to other organs, including liver, lung, and bone, so that the prognosis is generally poor [5,6]. LEC has been reported in many other organs, including the oral, oropharyngeal, nasal, and paranasal sinus LECs [7], and LEC involving the lung, skin, cervix, gallbladder, bladder, mammary gland, gastric mucosa, breast, lungs, liver, skin, urinary bladder, and cervix has also been reported [8,9,10,11,12,13,14]. EBV has been detected in some of these LECs, indicating that EBV may be involved in carcinogenesis, even in LELC of many organs [3,4,10,11,12,13,14]. Other researchers have argued that EBV may also be associated with the metastasis of TLEC to these organs [3,4]. Primary thymic LEC (TLEC) is a rare tumor that is one of the subtypes of thymic carcinoma. Although there have been several reports of thymic carcinoma, including TLEC, there have been few reports on the analysis of TLEC alone, with the only large-scale report being a literature review of 33 case reports by Iezzoni in 1995 [1]; this review has also been conducted by accumulating case reports to date.

### 1.1. Epidemiology of TLEC

Primary thymic LEC is classified as one of the 12 subtypes of thymic carcinoma according to the 2015 WHO classification [1]. However, in the 2021 WHO classification, the name was changed to lymphoepithelial carcinoma, one of the 14 subtypes of thymic carcinoma. Thymic carcinoma is also relatively rare, accounting for about 14% of thymic epithelial tumors, of which the frequency of TLEC is reported to be about 1.3–6% of thymic carcinomas [2]. Squamous cell carcinoma is the most common histological type of thymic carcinoma, followed by TLEC. In the database of the International Thymic Malignancy Interest Group (ITMIG), only 36 (0.59%) out of 6097 thymic epithelial tumors, including mainly thymoma and thymic carcinoma, were TLEC [15]. In the ITMIG database, squamous cell carcinoma accounts for approximately 80% of thymic carcinomas, while TLEC accounts for 6% [15]. The Japanese Association for Research on Thymus (JART), which maintains a database of thymic tumors in Japan, reports that TLEC accounts for 1.3% of thymic tumors [16]. Our facility has experienced 47 thymic carcinomas, of which only two were TLEC, which have already been reported, at a rate of 4.3% [17]. The ages of individuals affected by TLEC ranges from 4 to 76 years (median: 41 years), and they tend to have a bimodal peak incidence at 14 years and 48 years. Males are more commonly affected than female patients, with a male to female ratio of 2:1 [1,2].

Although NPC and primary lung LELC have been reported to be common in Asia, especially in South Asia [18], TLEC has also been reported in Europe and the United States, and no regional differences have been observed.

### 1.2. Diagnostic Strategy

Symptoms mainly depend on the stage of the disease, which includes chest pain, cough, fever, dyspnea, and fatigue. Patients with TLEC tend to present with the following symptoms due to the presence of a mediastinal mass effect: dull chest pain, cough, and difficulty in breathing [1,2]. Cases in advanced stages such as III and IV may be accompanied by invasion of surrounding organs and tissues such as the lung, pericardium, and diaphragm, and many cases of infiltration of the major vessels such as the superior vena cava, pulmonary artery, and aorta were also reported, with some cases accompanied by superior vena cava syndrome [19,20,21,22,23]. However, in some cases, the condition may be asymptomatic and may occur as an incidental anterior mediastinal mass found during routine imaging studies. Rarely, it may be complicated by the co-morbid occurrence of hypertrophic osteoarthropathy [24,25,26], polymyositis [1,2], or nephrotic syndrome [27,28], and systemic lupus erythematosus [26] in young patients. No association has been observed between TLEC and autoimmune diseases like myasthenia gravis, pure red cell aplasia, and hypogammaglobulinemia [1,2].

TLEC tends to show local invasion of contiguous structures in the anterior mediastinum such as the pleura, lung, diaphragm, and pericardium [1,2]. The most frequently distant metastatic sites are the lung, bone, and liver, while the brain, ovaries, and adrenal glands have also been reported [20], suggesting that there are various organs that can develop distant metastases.

Chest computed tomography (CT) scans tend to show a large and highly aggressive anterior mediastinal mass with or without areas of necrosis, hemorrhage, calcification, or cyst formation, but it is difficult to distinguish it from other thymic epithelial tumors [1,2]. There may also be gross invasion of contiguous mediastinal structures and widespread involvement of distant intrathoracic sites. Areas of low attenuation in the CT-imaging of the mass may correspond to necrosis [2]. 18F-fluorodeoxyglucose-positron emission tomography (FDG-PET) has been reported to show strong accumulation in both primary and metastatic lesions, showing strong accumulation except for necrotic areas [17,29,30,31]. The maximum standardized uptake value (SUVmax) ranges from 6 to 10 in these cases [17,31]; however, thymic carcinoma also shows strong accumulation, so it is difficult to distinguish TLEC from other types of thymic carcinoma on FDG-PET. Magnetic resonance imaging (MRI) provides additional information about the internal characteristics of mediastinal tumors without contrast. High-grade malignant tumors, such as thymic carcinoma, show heterogeneous and high intensity on T2-weighted images. High-risk thymomas and thymic carcinomas have lower apparent diffusion coefficient (ADC) values than low-risk thymomas, and advanced stage III–IV tumors have lower ADC values than stage I–II tumors. The evaluation of macrovascular invasion is considered similar to that of CT [32]. However, there are no findings that are characteristic of TLEC on CT. 

### 1.3. Pathological Diagnosis

**Figure 1:**
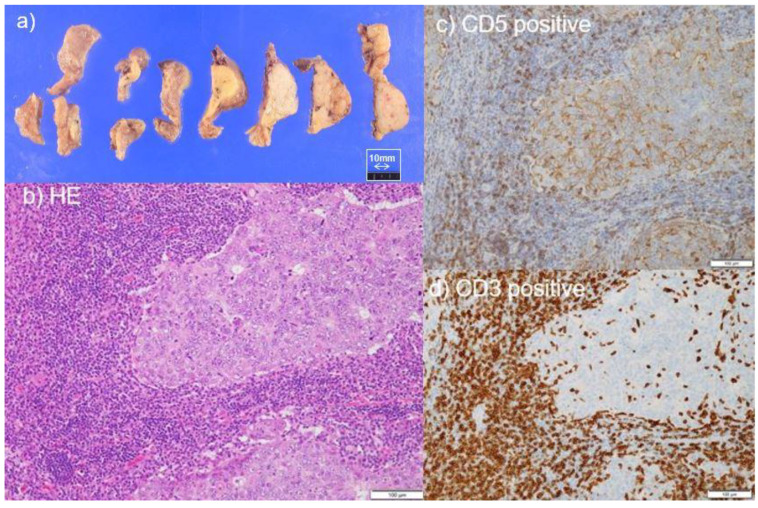
(**a**) Macroscopic appearance was yellowish-white necrotic areas. (**b**) The tumor cells were enlarged with large edematous nuclei and significant infiltration of lymphocytes in hematoxylin eosin staining. Immunostaining showed that the tumor cells were (**c**) CD5-positive and (**d**) the lymphocytes were CD3-positive.

The WHO classification is the standard diagnostic criterion for the pathological diagnosis of tumors of lung and thymus [1,2]. Macroscopically, the tumor surfaces of TLEC are solid and yellow–white because of necrosis (Figure 1a). Hematoxylin and eosin staining present with significant lymphocyte and plasma cell infiltration into the stroma and sheet-like growth foci with loose association of tumor cells (Figure 1b). Germinal centers, eosinophils, and granulomas may also be present. Undifferentiated carcinoma with syncytial appearing large tumor cells, with vesicular and distinct nucleoli, is classified as TLEC when EBV positivity is proven, even if the characteristic lymphocytic infiltration of LEC does not appear. On immunohistochemical examination, tumor cells were positive for pancytokeratin, cytokeratin (CK) 19, CK8, CK18, etc., which are positive in malignant tumors of glandular epithelium origin [1,2]. p63, a myoepithelial cell marker, was also highly positive. CD5 is another useful stain for diagnosis (Figure 1c). CD117 (c-kit) is variable, although many cases are positive. The Ki67 index is high in thymic carcinoma and has been reported to be high in TLEC, suggesting that TLEC is a highly malignant carcinoma [19]. It is occasionally difficult to differentiate TLEC from squamous cell carcinoma because squamous cell carcinoma can also show prominent lymphoplasmacytic infiltration. The absence of overt squamous differentiation in TLEC may help in the diagnosis, but it may be difficult in EBV-negative cases [1].

## 2. EBV Association

### 2.1. The Role of EBV in Neoplastic Diseases

The latency of EBV is classified into latency from 0 to 3, and the expression of virus-specific genes and proteins is restricted and different at each step [33]. In latent cells of healthy previously infected individuals, called Latency 0, only the EBV-encoded messenger RNA (EBER) and BamH1-A rightward reading frame transcript (BARTs) are expressed. Latency 1 has the least expression, Latency 2 has more expression than Latency1, and Latency 3 expresses all virus-specific genes and proteins.

When EBV is reactivated, the episome cleaves into a linear shape and is released into the extracellular space as infectious viral particles. The stage at which is process occurs is called the lytic/replicative phase. During this phase, more than 100 genes and their products are produced. Latency 1, which is found in Burkitt lymphoma, gastric cancer, and approximately two-thirds of nasopharyngeal carcinoma, displays the most restricted gene expression and is regulated by EBV-nuclear antigen 1 (EBNA1), EBER, and BARTs [34]. Latent membrane protein 2A is expressed in some cases. Latency 2, with the addition of the late membrane protein-1 (LMP1), is found in tumors that affect NK and T cells and is found in Hodgkin, NK/T, and pyothorax-associated lymphomas, in approximately one-third of nasopharyngeal carcinoma, and in gastric cancer. LMP1 positivity has been reported in TLEC [24,29,35,36]; therefore, TLEC is classified as Latency 2. The LMP1 and EBV nuclear antigen-2 (EBNA2) expressed during the lytic/replicative phase are oncogenic. In addition to the suppression of tumorigenesis by the regulation of LMP1 and EBNA, or the regulation of the expression of BZLF1 and other genes, epigenetic regulation mechanisms have been studied. LMP1 is essential for EBV-induced B cell immortalization [37]. In NPC, EBV virus capsid antigen IgG and EBV-DNase antibodies are elevated prior to carcinogenesis [38], which indicates that carcinogenesis is related to EBV reactivation. In Latency 3, all latent infection-related genes are expressed, and the dormant EBV-infected B cells are activated and proliferate uncontrollably due to immunodeficiency, mainly by cytotoxic T cells. This phenomenon is seen in post-transplant lymphoproliferative disorders and opportunistic lymphomas with acquired immunodeficiency syndrome.

EBER plays an important role in carcinogenesis because it is homologous to the adenovirus VA1 and VA2 genes and has similar RNA-dependent protein kinase binding properties. EBER has been shown to promote the growth of EBV-infected cells by inhibiting interferon-induced apoptosis and inducing the expression of growth factors such as interleukin (IL)-9 and insulin-like growth factor [39,40,41,42]. Burkitt lymphoma cells, an EBV-associated tumor, induce IL-10 by EBER, and proliferate using IL-10 as an autocrine self-growth factor [39]. The relationship between cytokines produced by EBV-infected cells and carcinogenesis has attracted much attention.

### 2.2. Proof of EBV Infection in Tissues

Polymerase chain reaction and southern blot hybridization (SB) methods have been used to search for EBV DNA. Although SB is more specific because of poor sensitivity, which increases the specificity and reliability of the test, it is not possible, for viral DNA verification, to identify whether the EBV genome is derived from tumor cells using this method. Therefore, in situ hybridization (ISH) using paraffin-embedded blocks is widely used to detect EBV viral infection in tumor cells. Infected cells that possess the EBV genome but do not produce viral particles are in a latent infection state, and it is known that such infected cells synthesize large amounts of two types of small RNAs encoded by the EB virus. These non-coding RNAs, which are not translated into proteins, consist of EBER1 and EBER2 of 167 and 173 nucleotides, respectively, and are mainly localized in the nucleus where they are present in high concentrations, up to 10^7^ copies per cell. They can be identified in the nuclei of individual tumor cells by ISH, even in paraffin-embedded tissues [1,2]. Since ISH can detect EBV genomes with high sensitivity and tissue specificity, it is widely used for the diagnosis of EBV-associated tumors [43]. The detection of EBER 1 by ISH in tumor cells indicates that EBERs are transcribed from the EBV genome as a result of latent EBV infection. Immunostaining for LMP1, a type of membrane protein produced by EBV, also provides evidence of EBV infection but is less sensitive than EBER-ISH.

EBV virus capsid antigen IgG antibodies, EBV IgA antibodies, EBV early antigen, diffuse type, and restricted type (EA-DR) antibodies are known to be elevated in Burkitt lymphoma, NPC, gastric cancer, and TLEC, which are EB virus-related tumors [44].

### 2.3. Association of EBV with Thymus, Thymic Epithelial Tumors, Thymic Carcinoma, and TLEC

The possible association of EBV with thymic carcinoma was first reported in 1982 by Wick et al. In 1985 [45], Lyvraz et al. reported EBV infection of TLEC tumor cells using SB and polymerase chain reaction [46]. In 1988, Dimery et al. also demonstrated EBV infection of TLEC using the SB method [47]. EBV infection has also been reported in thymic epithelial tumors other than thymic carcinoma and thymic hyperplasia associated with myasthenia gravis (MG) [21,48]. In 1988, McGuire et al. measured the EBV genome and proved the presence of the genome for thymic hyperplasia associated with thymoma and MG [21]. In this report, since one of the three thymomas had features of TLEC, it was suggested that TLEC may have been derived from a thymoma. This case is now classified as TLEC, while the two other cases are histopathologically thymoma. This means the EBV genome was present in thymoma cells. The presence of the EBV genome in thymic hyperplasia was also demonstrated for the first time in this report, in patients with both coexisting and non-coexisting MG [21]. EBV has been associated with the development of autoimmune diseases, including multiple sclerosis, systemic lupus erythematosus, and rheumatoid arthritis [49]; in MG, EBER-positive cells have been found in thymic follicular hyperplasia and diffuse hyperplasia, and LMP1-positive cases have also been reported [48]. 

In the thymus of MG patients, a high percentage of tissue-infiltrating B cells harboring EBV form EBV-rich ectopic lymphoid tissue. Since B cells are usually present in the thymus, the possibility that EBV infection occurs directly in the thymus and unknown factors may induce local viral reactivation.

Although it is unclear whether EBV is associated with the pathogenesis of MG or thymoma with MG, when considered in conjunction with reports of EBV infection in MG-associated thymomas and TLEC [21], Cavalcante et al. have suggested that active EBV infection of the MG thymus may increase the risk of malignant transformation of lymphoid and epithelial cell components [48].

Zhang et al. reported a systematic review of 22 studies on the association between thymic epithelial tumors and EBV [50]. According to the results of these reports, EBV positivity in thymic epithelial tumors varies widely among reports, but the pooled estimated incidence of EBV positivity was 9% (95% confidence interval (CI), 1–23%) for thymic epithelial tumors without MG, 20% (95% CI, 0–54%) for thymic epithelial tumors with MG, 6% (95% CI, 0–54%), and 6% (95% CI, 0–21%) for thymic carcinoma. However, when limited to TLEC among thymic carcinomas, 12 of 23 cases (52.2%) were EBV-positive. It can be said that EBV is also present in thymic epithelial tumors, but the low prevalence of EBV positivity suggests that it plays a minor role in the pathogenesis of thymoma. Even in thymic carcinoma, the very low EBV-positive cases, except in TLEC, suggest that EBV plays a minor role in tumorigenesis. However, when limited to TLEC, the positive rate increases to approximately 50%, and EBV plays an important role in carcinogens in addition to other causes. As there have been reports of TLEC with thymomatous component [51] and type B3 thymoma [52], it has been suggested that TLEC may be transformed from thymoma [51], but this is speculative, and the mechanism of carcinogenesis remains to be elucidated.

## 3. Review of the Literature

In this retrospective literature review, we searched for 58 cases in 34 reports with age and gender described, from 1985, when Leyvraz et al. first proved the presence of the EBV genome in TLEC [46], to 2021. Details of these cases from the literature are shown in Table 1 [17,20,21,22,23,24,25,26,27,28,29,30,31,35,36,44,46,47,52,53,54,55,56,57,58,59,60,61,62,63,64,65,66]. We obtained data on the listed author of the publication, the stage according to the Masaoka classification, EBV detection method, EBV-positivity, treatment, and prognosis, and analyzed the results.

Since TLEC is a rare histological type of thymic carcinoma, patient background, treatment strategy, and prognosis are mostly based on case reports.

The mean age of patients from 36 cases in the ITMIG database was reported to be 49 years [15], but these 36 cases were not included in this analysis, as detailed data on individual cases were not available. In 58 cases in Table 1, the mean age was 40.5 ± 22.6 years, but many cases were reported in younger patients such as teenagers. The peak age was bimodal, ranging from the teens to the 60s, with peak values of 16.8 and 60.3 years. The male-to-female ratio was 1.52:1, which was lower than that of squamous cell carcinoma, the most common type of thymic carcinoma (mean age, 48.5 ± 19.3; male-to-female ratio, 1.25:1) [1,2]; the proportion of men was slightly higher. Of those patients whose stage information was provided, 30.2% were Masaoka classification I/II, 14.0% were III, and 55.8% were IV. Of the 36 ITMIG cases, 27.5% were Masaoka I/II, 26.9% were III, and 44.4% were IV, indicating that most of the patients were found to have advanced stage IV with pleural dissemination, malignant pleural effusion, lymph node metastasis, and distant metastasis. Lymph node metastasis was the most common cause of stage IVb disease.

### 3.1. Relevance to EBV in TLEC in the Reported Cases

As shown in Table 1, 46 of 58 cases were searched for EBV infection, of which 20 (43.5%) were positive. In the 1980s, the search was mainly performed by SB, but recently, ISH has been the most common method. The WHO classification indicated that only about 50% of TLEC cases were EBV-positive [1,2], of which the positive rate was similar in this review, which included cases to date. The EBV positivity rate in TELC is much lower than in NPC, where more than 90% of tumors are positive [67], or in EBV-associated gastric cancer, where 80–90% are EBV-positive. However, since EBV positivity is much lower in other histological subtypes of thymic carcinoma [49], EBV infection is considered to have an important impact on the etiology of TLEC among thymic carcinoma subtypes. Therefore, even tumors classified as undifferentiated types, which do not show histopathological images such as marked infiltration of lymphocytes characteristic of LEC, are classified as TLEC if they are EBV-positive [1,2]. However, the low positivity rate suggests that EBV is not the only cause of the disease.

### 3.2. Survival

#### 3.2.1. Statistical Analysis

Values ± the standard deviation were presented. Comparisons between 2 groups were made using a Mann–Whitney U test, with a chi-square test used for categorical variables. Overall survival rate was calculated from the data of follow-up period until the time of death from any cause or last visit (censored OS) using the Kaplan–Meier method. The overall survival rate was calculated from the data of the follow-up period until the time of death from any cause or last visit (censored OS) using the Kaplan–Meier method. A log-rank test was used to assess differences between subgroups. A probability value <0.05 was considered significant. All analyses were performed using the JMP 15.1.0 statistical software package (SAS Institute Inc., Cary, NC, USA).

#### 3.2.2. TLEC Survival

**Figure 2:**
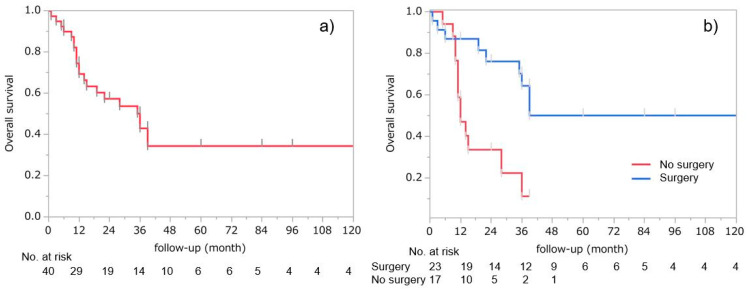
(**a**) Two-year survival rates were 57.4%, and 5-year survival rate was 34.4% in 40 cases. (**b**) Among the 30 patients whose EBV status and prognosis were stated, the 2-year survival rate was 36.0% for positive cases and 58.3% for negative cases; however, the difference was not significant.

TLEC is a highly malignant tumor with a poor prognosis; Iezzoni et al. reported that in a review of 33 cases, 29 (88%) died of the primary disease, with a mean survival of 16 months [1]. This report included all reports before 1993, which also overlapped in this review. Following this, improvements in treatment methods may have had an impact. In the WHO classification described a median survival time (MST) of 36 months and a survival rate of approximately 60% [1,2]. In 40 cases in this review, the median follow-up period was 36 months (1–192 months), and the MST was 22 months; the 2-year survival rate was 57.4%, and the 5-year survival rate was 34.4% (Figure 2a).

The presence or absence of EBV infection is not thought to affect prognosis, based only on Iezzoni’s report that four out of five (80%) EBV-positive cases died at 11, 14, 22, and 39 months [44,46,59], with evidence that survival did not differ from negative cases [1]. In the 32 cases, including 15 positive and 17 negative in which EBV examination was performed and the results were clearly stated, the number of deaths was nine (60.0%) with positive results and six (35.3%) with negative, and the number of deaths was higher in the positive cases, but the difference was not statistically significant (*p* = 0.29). Furthermore, among the 30 patients whose EBV status and prognosis were stated, the 2-year survival rate was 36.0% for positive cases and 58.3% for negative cases, which was lower for positive cases; however, the difference was not significant (*p* = 0.31) (Figure 2b). Presently, the presence or absence of EBV positivity does not seem to have an effect on prognosis.

### 3.3. Treatment

In thymic carcinoma, Ahmad et al. reported that complete surgical resection was an important prognostic factor, and that postoperative radiotherapy contributed to improved prognosis, while chemotherapy failed to show significance [68]; Ruffini et al. recommended surgical resection whenever possible [69]. Hishida et al. reported that complete resection was an independent prognostic factor for overall survival, and that incomplete resection had a better prognosis than non-resection [16]. These large database reports of thymic carcinoma also included 40 (6%) [68], 5 (4%) [69], and 4 (1.3%) [16] cases of TLEC, respectively, but the most common type was squamous cell carcinoma, and there was no analysis of TLEC alone. Ahmad et al. also found no significant differences between the histological subtypes and overall survival. However, this association could not be demonstrated due to the small number of cases other than squamous cell carcinoma; further prospective studies are needed [68]. Since TLEC is a relatively large proportion of rare histological types other than squamous cell carcinoma, complete resection may improve the prognosis if it is considered equivalent to squamous cell carcinoma.

A total of 23 patients who underwent surgical treatment and 17 patients who did not undergo resection were compared to 40 patients who were described for the treatment, follow-up period, of which prognosis had been reported. The 5-year survival rate was 50.1% in the surgical treatment group and 11.2% in the no surgical treatment group, which was significantly higher in the surgical treatment group (*p* = 0.0029). Of the eight patients who underwent surgery alone, six had a long-term survival of more than 3 years [7,51,60,63]. In particular, three cases by Suster et al. showed a very long-term survival of 8 and 16 years [51].

A 67-year-old woman with stage II Masaoka disease, who underwent total thymectomy and anterior mediastinal lymph node dissection for complete resection and no adjuvant therapy was administered, is still under observation and has been recurrence-free for 4 years [17]. In addition, 6 patients survived among 12 cases of multimodal treatment with surgery, chemotherapy, and radiation therapy. Although the number of cases is too small to make a comparison, it can be assumed that the prognosis can be improved by multidisciplinary treatment, including surgery, when surgical treatment is possible, or at least when there is no distant metastasis.

**Figure 3:**
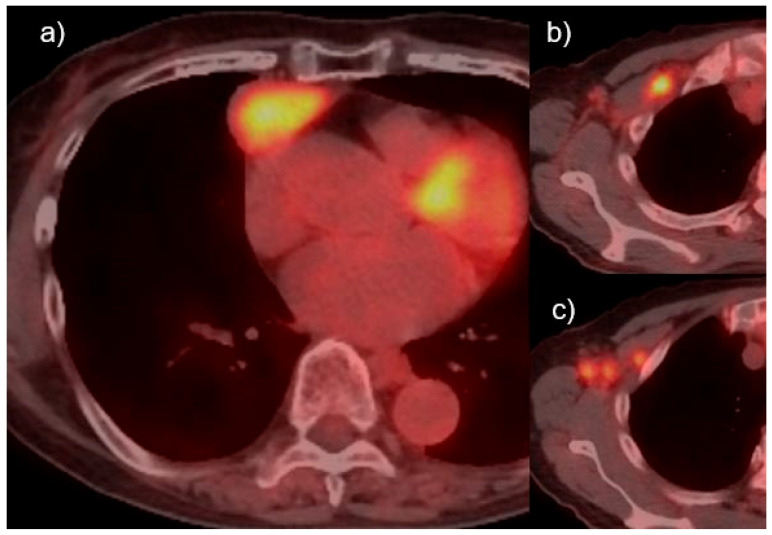
(**a**) An 81-year-old woman case. Anterior mediastinal tumor was shown in a 58 × 45 × 26 mm with fluorodeoxyglucose (FDG) accumulation (maximum standardized uptake value (SUVmax) 6.5) on fluorodeoxyglucose-positron emission tomography (FDG-PET). (**b**) After 5 months, right supraclavicular fossa and (**c**) right axially lymph nodes diagnosed metastasis were revealed with high FDG accumulation (SUVmax 6.6).

However, even in the six cases with complete resection and prognosis, two cases had recurrences [17,51]. One patient was an 81-year-old woman who had no symptoms, and an anterior mediastinal tumor was shown in a 58 × 45 × 26 mm CT and FDG accumulation (SUVmax 6.5) on FDG-PET (Figure 3a); she underwent total thymectomy and anterior mediastinal lymph node dissection. The tumor was substantial, with some yellowish-white necrotic areas (Figure 1a). Tumor cells were enlarged with large edematous nuclei and significant infiltration of lymphocytes (Figure 1b), and immunostaining showed that the tumor cells were bcl-, CD5-, and p40-positive (Figure 1c), and the lymphocytes were CD3-positive (Figure 1d) and CD99-negative, leading to the diagnosis of TLEC. EBER-ISH was negative. After 5 months, chest CT showed enlarged lymph nodes in the right supraclavicular fossa, right axilla, right lower neck, and mediastinum, and PET–CT showed FDG accumulation (SUVmax 6.6) in the right supraclavicular fossa (Figure 3b) and right axially lymph nodes (Figure 3c). A needle biopsy of the right axillary lymph node was performed, and a diagnosis of axially lymph node metastasis of TLEC was made. Thus, even if complete resection is achieved at a relatively early stage, recurrence may occur in a short period of time, and a careful follow-up is necessary on a regular basis. 

Until the 2000s, the drugs for regimens used for lymphoma and thymoma, including ifosfamide + cisplatin (CDDP) + etoposide (VP-16) (ICE therapy), cyclophosphamide (CPA), and cisplatin (CDDP) + etoposide (VP-16) + vincristine (VCR) + predonizolone (P) (CVP therapy) for lymphoma, adriamycin (ADR) + CDDP + VCR + CPA (ADOC therapy) for thymoma, and CDDP + doxorubicin (DXR) + methylprednisolone (MP) (CAMP therapy), were often used. The reported regimens were as follows: Bleomysin + DXR + CDDP + P [46], CPA + VCR + methotrexate + DXR + cis-diamine dichloroplatinum [53], CDDP + DXR [22], VCR + CPA [25], vinblastine + CDDP + ADR + bleomycin + P [61], ifosfamide + VCR + dactinomycin, CDDP + VP-16 [26], ICE [36,61], CDDP + VP-16 + DXR [64], and CDDP + DXR + VCR + CPA [28]. Subsequently, around 2010, the number of cases performed based on the treatment of NPC increased. In NPC, combination chemotherapy with carboplatin (CBDCA) and paclitaxel (PTX) is the first-line regimen. The efficacy of CBDCA and PTX has also been demonstrated in advanced thymic carcinoma [70], which has been used in recent reports. Reported regimens included CBDCA + PTX [35], CBDCA + nanoparticle albumin-bound paclitaxel [30], and docetaxel, CDDP + VP-16 [25].

In addition, many patients have advanced cancers; therefore, due to their poor performance status, surgery and chemotherapy are difficult to administer, and as a result, radiotherapy was first introduced in many cases. However, at present, there are no reports on the outcomes of chemotherapy, radiotherapy, and surgery, either alone or in combination, and they have yet to be established as standard treatment methods. 

It has been reported that programmed death-ligand 1(PD-L1) is highly expressed in half the cases of primary lung LELC, which is one of the EBV-related cancers [71]. Pembrolizumab is effective in the treatment of patients with thymic epithelial tumors, including thymic carcinoma that expresses high levels of PD-L1 [72]. Although there are no reports about PD-L1 expression in TLEC, it is expected that it would be worthwhile to evaluate PD-L1 expression in future cases.

### 3.4. Prognosis

TLEC is a highly malignant tumor with a poor prognosis and relatively low survival rate of 16 months in 88% of patients [1,2]. The survival rates analyzed from 40 cases for which the follow-up period and prognosis were described in previous reports were also low. EBV positivity status does not seem to have an effect on prognosis, and their survival and recurrence rates are similar to those of other types of thymic cancers [1,2].

## 4. Conclusions

TLEC demonstrated EBV infection in approximately half the cases. The low rate of EBV positivity in thymic epithelial tumors, such as thymoma and other histologic types of thymic carcinoma, suggests that EBV infection may contribute to carcinogenesis in TLEC. However, the prognosis is poor because TLEC is often detected at an advanced stage; a multimodality treatment, including surgery, may improve the prognosis, and as TLEC is a rare tumor, further accumulation of cases is necessary.

## Figures and Tables

**Table 1:** Details of reported case.

Author	Year	Ref.	Age	Sex	Masaoka Stage	EBV		Treatment	Follow-Up (Month)	Prognosis
Leyvraz	1985	[46]	19	M	III	SB *	+	CRT ^¶^	11	Dead
Taylor	1988	[22]	43	F	IVb	N/A ^†^		RT ** + CTx ^††^	28	Dead
			28	M	IVb	N/A		RT + CTx	36	Dead
Dimery	1988	[47]	30	F	IVb	SB *	+	RT + CTx	24	Alive
McGuire	1988	[21]	73	F	N/A	SB	+	S ^‡‡^	N/A	N/A
Harutmann	1990	[20]	38	F	IVb	ISH ^‡^	−	RT + CTx	5	Dead
(Niedbeck)		[54]	68	M	N/A	ISH	−	S + RT	3	Dead
			46	M	IVb	ISH	−	RT	9	Dead
Kuo	1990	[55]	19	M	IVa	N/A		S + CRT	39	Dead
			41	M	IVb	N/A		S + CRT	19	Dead
Matsuno	1992	[56]	10	M	N/A	PCR ^§^	+	CRT	14	Dead
Mann	1992	[57]	26	F	N/A	ISH	+	N/A	N/A	N/A
			71	M	N/A	ISH	−	N/A	N/A	N/A
			48	M	N/A	ISH	−	N/A	N/A	N/A
			35	M	N/A	ISH	−	N/A	N/A	N/A
Patton	1993	[58]	15	M	IVb	SB	+	CRT	12	Alive
Wu	1993	[59]	19	M	N/A	ISH	+	S + CRT	39	Dead
			42	M	N/A	ISH	−	N/A	N/A	N/A
			68	F	N/A	ISH	−	N/A	N/A	N/A
			75	M	N/A	ISH	−	N/A	N/A	N/A
			37	F	N/A	ISH	−	N/A	N/A	N/A
Fujii	1993	[44]	13	F	IVb	ISH	+	S + CRT	22	Dead
Ilhan	1994	[25]	13	F	IVb	N/A		S + CRT	N/A	Alive
Niehues	1996	[27]	14	M	N/A	ISH	+	S + CRT	12	Alive
Hsu	1998	[23]	65	F	N/A	N/A		S + RT	6	Alive
Takahashi	2000	[60]	66	F	III	N/A		S	35	Dead
Cataldo	2000	[26]	11	M	IVb	ISH	+	CRT	12	Dead
Stephan	2000	[61]	12	F	IVb	ISH	+	CRT	12	Dead
Nicholato	2001	[62]	55	M	IV	N/A		CTx	39	Alive
Tateyama	2001	[63]	59	M	IVa	ISH	+	S	39	Alive
			56	F	III	ISH	−	S	N/A	N/A
Hsueh	2006	[24]	14	M	IVb	ISH	+	RT	10	Dead
			10	M	IVb	ISH, LMP-1 ^||^	+	RT	11	Dead
Yaris	2006	[64]	16	F	IVb	N/A		CRT	15	Dead
Tacylidiz	2007	[36]	10	M	III	LMP-1	+	S + CRT	12	Alive
Kilis-Pstrusinska	2008	[28]	16	M	IVa	N/A		CTx	11	Dead
Koppula	2009	[29]	24	M	IVb	LMP-1	+	CRT	N/A	N/A
Januskiewicz	2012	[65]	17	M	IVb	PCR	+	N/A	N/A	N/A
Sekihara	2014	[35]	14	M	IVb	LMP-1	+	CTx	10	Dead
Shima	2016	[30]	22	M	Iva	ISH	+	CRT	N/A	N/A
Suster	2018	[52]	56	M	I	ISH	−	S ^§§^	96	Alive
			55	M	II	ISH	−	S	192	Alive
			47	F	IV	ISH	−	S ^§§^	N/A	Alive
			57	F	II	ISH	−	S	192	Alive
			55	M	I	ISH	−	S + RT	96	Alive
			48	M	N/A	ISH	−	S ^§§^	N/A	N/A
			51	F	I	ISH	−	S ^§§^	36	Dead
			61	F	I	ISH	−	S ^§§^	60	Alive
			60	M	II	ISH	−	S	84	Alive
			64	M	I	ISH	−	S + RT	N/A	Alive
			20	M	IV	ISH	+	S + CRT	12	Alive
			67	F	III	ISH	−	S	1	Dead
Pan	2019	[66]	7	M	IVb	N/A		S + CRT	N/A	N/A
Kawagishi	2019	[17]	65	F	II	ISH	−	S	36	Alive
			81	F	II	ISH	−	S	6	Dead
Fujita	2019	[31]	81	F	III	ISH	−	RT	24	Alive
Guan	2021	[53]	64	F	I or II	N/A		S + CTx	36	Alive
			52	M	I or II	ISH	−	S + CRT	24	Alive

SB *; southern blot, N/A ^†^; not available, ISH ^‡^; in situ hybridization, LMP-1 ^||^; late membrane protein-1, PCR ^§^; polymerase chain reaction, CRT ^¶^; chemoradiotherapy, CTx **; chemotherapy, RT ^††^; radiotherapy, S ^‡‡^; surgery only, S ^§§^; adjuvant therapy was unknown.

## Data Availability

All data generated or analyzed during this study are included in this published article.
